# Functional Characterization of *FgAsp*, a Gene Coding an Aspartic Acid Protease in *Fusarium graminearum*

**DOI:** 10.3390/jof10120879

**Published:** 2024-12-17

**Authors:** Ping Li, Zhizhen Fu, Mengru Wang, Tian Yang, Yan Li, Dongfang Ma

**Affiliations:** Key Laboratory of Sustainable Crop Production in the Middle Reaches of the Yangtze River, College of Agriculture, Yangtze University, Jingzhou 434025, China; 2022710831@yangtzeu.edu.cn (P.L.); 2022720883@yangtzeu.edu.cn (Z.F.); 2021720804@yangtzeu.edu.cn (M.W.); 2022004773@yangtzeu.edu.cn (T.Y.); yan_li@yangtzeu.edu.cn (Y.L.)

**Keywords:** *Fusarium graminearum*, aspartic proteases, gene knockout, pathogenicity

## Abstract

Aspartic proteases (APs), hydrolases with aspartic acid residues as catalytic active sites, are closely associated with processes such as plant growth and development and fungal and bacterial pathogenesis. *F. graminearum* is the dominant pathogenic fungus that causes Fusarium head blight (FHB) in wheat. However, the relationship of APs to the growth, development, and pathogenesis of *F*. *graminearum* is not clear. Therefore, we selected the *FGSG_09558* gene, whose function annotation is aspartate protease, for further study. In this study, *FGSG_09558* was found to contain a conserved structural domain and signal peptide sequence of aspartic acid protease and was therefore named *FgAsp*. The function of *FgAsp* in *F. graminearum* was investigated by constructing the knockout and complementation mutants of this gene. The results showed that with respect to the wild type (PH-1), the knockout mutant showed a significant reduction in mycelial growth, asexual spore production, and sexual spore formation, highlighting the key role of *FgAsp* in the growth and development of *F. graminearum*. In addition, the mutants showed a significant reduction in the virulence and accumulation level of deoxynivalenol (DON) content on maize whiskers, wheat germ sheaths, and wheat ears. DON, as a key factor of virulence, plays an important role in the *F. graminearum* infection of wheat ears, suggesting that *FgAsp* is involved in the regulation of *F. graminearum* pathogenicity by affecting the accumulation of the DON toxin. *FgAsp* had a significant effect on the ability of *F. graminearum* to utilize various sugars, especially arabinose. In response to the stress, hydrogen peroxide inhibited the growth of the mutant most significantly, indicating the important function of *FgAsp* in the strain’s response to environmental stress. Finally, *FgAsp* plays a key role in the regulation of *F. graminearum* growth and development, pathogenicity, and environmental stress response.

## 1. Introduction

*Fusarium graminearum* is a phytopathogenic fungus responsible for wheat scab [1]. In wheat plants, it infects the spike, leading to the contamination of mature seeds by mycotoxins such as deoxynivalenol (DON) [2,3]. These mycotoxins adversely affect the health of both humans and livestock [4,5,6,7]. *Fusarium graminearum* thrives in warm and humid environments and can infect various gramineous plants, causing diseases like wheat blight. It is widely distributed around the globe and contributes to significant economic losses in agriculture [8,9]. Preventive and management strategies for *F. graminearum* include the use of resistant plant varieties and the application of fungicides [8,9,10]. Research into the molecular biology and genetics of *F. graminearum* has provided valuable insights into the fungus’s biology, pathogenic mechanisms, and interactions with host crops. The application of gene knockout technology allows the targeted deletion of specific genes, thereby elucidating their functions in biological characterization.

Aspartic proteinases (APs) are a class of hydrolytic proteases that maintain high activity under acidic conditions. These enzymes are found in plants, animals, and microorganisms. Structurally, they exhibit a bilobal fold and contain a highly conserved DT/SG sequence (Asp-Gly-Thr/Ser). Additionally, they possess an aspartic acid residue at both the amino and carboxyl termini [11,12,13]. In the cell, APs utilize the aspartic acid residues in their active sites, making them important targets for research into the development of fungicides [14]. A gene encoding an aspartic proteinase cloned from *Trichoderma asperellum* (ACCC30536) was successfully expressed in *Escherichia coli* BL21. The purified recombinant protein, *rAsp55*, demonstrated significant protease activity and was effective in inhibiting the mycelial growth of the phytopathogenic fungus *Alternaria alternata* [15].

APs play important roles in various biological processes in fungi, including biofilm development, trophic hyphae formation, spore development, host adhesion and invasion, and immune evasion. Among fungal pathogens, *Candida albicans* is particularly notable, as its secreted aspartic proteases (SAPs) are key factors contributing to the virulence of pathogenic fungal infections. The aspartic proteases from the *Candida* genus exploit their hydrolase activity to extract nutrients from the environment, reduce host defenses, and disrupt the internal homeostasis of the human host [16]. In *C. albicans*, the hydrolytic cleavage of mucin Msb2 by the aspartic protease SAP8 activates the Cek1-MAPK pathway, which induces cell morphogenesis, biofilm formation, and fungal budding and enhances the pathogen’s ability to infect the host [17]. Additionally, the aspartic protease SAP9 regulates mycelial formation by modulating EFG1 expression through a cAMP-dependent pathway [18]. Furthermore, the deletion of Sapa3, the major secreted aspartyl protease in *C. auris*, results in a significant decrease in SAP activity and a marked reduction in its virulence [19]. Similar roles are observed in aspartic proteases secreted by other filamentous fungi. For example, the aspartic protease *P6281* from *Trichoderma harzianum* is involved in its biological control activity. It plays a role in the parasitism of phytopathogenic fungi and can inhibit spore germination and the growth of plant and animal pathogens, including *Botrytis cinerea*, *Mucor circinelloides*, *Aspergillus fumigatus*, *A. flavus*, *Rhizoctonia solani*, and *C. albicans* [20]. Moreover, the aspartic protease FolAsp enhances virulence expression in the fungus *Fusarium oxysporum f.* sp. *lycopersici* while inhibiting reactive oxygen species (ROS) production in plants [21]. Studies have also reported genes involved in aspartic protease synthesis in *F. graminearum*. Notably, the barley renin-like aspartic protease *HvNEP-1* has been shown to degrade Fusarium phytase, affect toxin production, and inhibit fungal growth [22]. Additionally, the *F. graminearum* effector protease *FgTPP1* enhances pathogen virulence by attenuating the chitin-mediated activation of mitogen-activated protein kinase (MAPK) signaling, ROS production, and evasion of host immune responses through the expression of auto-antioxidant proteins when expressed in plants [23].

Considering the importance of secreted aspartyl proteases in various organisms and their potential as therapeutic targets, this study aims to functionally analyze *FgAsp* (FGSG_09558), a gene annotated as secreted aspartyl proteases in the *F. graminearum* genome. We obtained knockout and complementary mutants to investigate the functional roles of *FgAsp* in mycelial growth, asexual reproduction, sexual reproduction, pathogenicity, and stress responses in *F. graminearum*. According to the results obtained, the FgAsp gene represents a new target for developing plant protection means for *F. graminearum* management.

## 2. Materials and Methods

### 2.1. Screening for the Target Gene

A total of 200 genes were identified by predicting signal peptides from the *F. graminearum* genome database (http://fgbase.wheatscab.com/, accessed on 20 September 2023) using the SignalP-6.0 tool (https://services.healthtech.dtu.dk/services/SignalP-6.0/, accessed on 20 September 2023). Subsequently, transmembrane structures were predicted for the genes containing signal peptides using TMHMM-2.0 (https://services.healthtech.dtu.dk/services/TMHMM-2.0/, accessed on 20 September 2023). The identified genes were then subjected to functional annotation using BLAST on NCBI (https://www.ncbi.nlm.nih.gov/, accessed on 20 September 2023). Ultimately, we selected the gene functionally annotated as aspartic protease FGSG_09558 for further study.

### 2.2. Bioinformatics Analysis

The genome data for *F. graminearum* were retrieved from the *F. graminearum* genome database (http://fgbase.wheatscab.com/, accessed on 20 September 2023). Conserved domains within the *FgAsp* (*FGSG_09558*) gene were identified using the online Pfam tool (http://pfam.xfam.org/, accessed on 20 September 2023) [24]. The tertiary structure models of FgAsp proteins were generated using SWISS-MODEL software (https://swissmodel.expasy.org/, accessed on 20 September 2023) [25]. The transmembrane structure of *FgAsp* was predicted via the TMHMM-2.0 prediction website (https://services.healthtech.dtu.dk/services/TMHMM-2.0/, accessed on 20 September 2023) [26]. Predicted expression data for the *FgAsp* gene were downloaded from the *F. graminearum* genome database (http://fgbase.wheatscab.com/, accessed on 20 September 2023) and analyzed using Prism-8.0 software to process and interpret the expression levels [27]. The SignalP-6.0 server (https://services.healthtech.dtu.dk/services/SignalP-6.0/, accessed on 20 September 2023) was utilized to identify the presence and cleavage sites of signal peptides in FgAsp proteins [28]. Predictions for subcellular localization were conducted using the Plant-PLoc server (http://www.csbio.sjtu.edu.cn/bioinf/plant/, accessed on 20 September 2023).

### 2.3. Split PCR Construction of Gene Knockout Cassette

Primer Premier 5.0 was utilized to design primers specific to the *FgAsp* gene (Table S1). Genomic DNA from the PH-1 strain served as the template for amplifying the upstream L1 (1473 bp) fragment and the downstream L2 (1372 bp) fragment of *FgAsp* using the primer pairs FgAsp-1F/2R and FgAsp-3F/4R. The selectable marker gene hygromycin B phosphotransferase (*hph*), present in the plasmid pCB1003, was employed for gene replacement. The upstream H1 (763 bp) region and downstream H2 (929 bp) DNA fragment of the *hph* gene were amplified with the primer pairs HYG/F/YG/R and HY/F/HYG/R. The upstream L1 and H1 fragments served as template 1, while the downstream L2 and H2 fragments served as template 2. The fusion fragments FgAsp-LH1 (2236 bp) and FgAsp-HL2 (2301 bp), which formed the knockout cassette replacing the target gene with the *hph* gene, were amplified through overlap PCR using the FgAsp-1F/YG/R and HY/F/FgAsp-4R primers, respectively (Figure S1) [29].

### 2.4. Gene Knockout and Complementation

**Figure 1:**
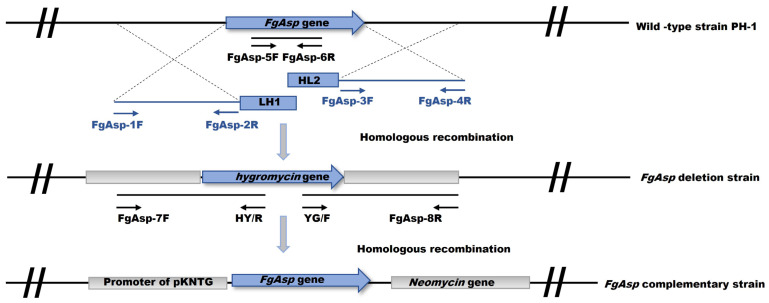
The *FgAsp* gene deletion and complementation strategies.

The PEG-mediated protoplast transformation method was employed for gene knockout (Figure 1) [30]. Protoplast preparation from PH-1 involved collecting young mycelia from YEPD liquid culture medium after 12 h. The mycelia were filtered using aseptic filter paper, washed twice with sterile water, and then washed once with 1.2 M KCl to maintain osmotic pressure. The pre-configured enzymolysis solution buffer, containing 2% lysozyme, 3% snailase, 2% cellulase, and 0.5% lysozyme in 10 mL KCl, was applied to the mycelium for 3 h. Finally, four layers of sterile microscope wipes with one layer of sterile filter cloth were used to filter the mycelial enzymatic mixture. The filtrate was collected into a 50 mL aseptic centrifuge tube at 4000 rpm for 8 min. The supernatant was quickly discarded, and 1 mL of STC buffer liquid (0.735% CaCl_2_·2H_2_O (Sinopharm, Shanghai, China), 10% 0.5 M Tris-Cl (pH 8.0), 20% sucrose (Sinopharm, Shanghai, China)) was added to the precipitate to resuspend PH-1 protoplasts.

A total of 10 µg DNA (FgAsp-LH1 and FgAsp-HL2) was added to 500 µL of PH-1 protoplasts for transformation proportionately. Subsequently, 1 mL PTC buffer (40% PEG8000 (Sigma-Aldrich, St. Louis, MO, USA) in STC buffer) was added gently, and TB3 liquid medium was added to 7.5 mL and incubated at 110 rpm at 25 °C for 12 to 16 h for transformation in dark. The regenerating protoplasts were mixed with the bottom layer TB3 agar melted medium with 50 µg/mL of ampicillin and 200 µg/mL of hygromycin B and then screened by top layer containing 250 µg/mL of hygromycin B incubated at 25 °C for 24–72 h.

Four pairs of primers, FgAsp-5F/6R, H850/H852, FgAsp-7F/H855R, and H856F/FgAsp-8R (Table S1), were utilized for screening of potential transformants. FgAsp-5F/6R and H850/H852 to detect the presence of the *FgAsp* gene and *hph* gene, respectively, and FgAsp-7F/H855R and H856F/FgAsp-8R to detect the completion of homologous recombination in the upstream and downstream regions, respectively. The deletion of the target gene, the presence of the *hph* gene, and the success of homologous recombination in upstream and downstream regions together indicate positive transformants. Additionally, positive transformants were confirmed by RT-PCR, where the cDNA of PH-1 and positive transformants was amplified using RT-PCR-FgAspF/R with target gene primers (the Actin gene served as the internal reference). If the Actin gene is amplified in both PH-1 and positive invertors, but the target gene is amplified only in PH-1, this indicates that the *FgAsp* gene has been successfully replaced by the hygromycin resistance gene, confirming that the positive invertor is the knockout mutant Δ*FgAsp*.

In the gene complementation assay, PH-1 genomic DNA served as the template, with primers CMFgAsp-F and CMFgAsp-R (Table S1), along with high-fidelity enzymes, used to amplify gene fragments containing the promoter and no stop codon. The pKNT-FgAsp-GFP chimeric complementation vector was then ligated into the pKNT-GFP (neomycin gene-containing) plasmid and transformed into DH5α receptor cells. This complementary vector was subsequently transferred into Δ*FgAsp* protoplasts via PEG-mediated transformation, and the transformants were selected using 200 mg/mL G418 and identified by PCR using the primers FgAsp-CMF/R (Table S1). The confirmed complementary strain CΔ*FgAsp* was used for subsequent experiments.

### 2.5. Phenotypic Identification and Conidial Formation

The strains were activated on potato sucrose agar (PSA) medium (Table S3) for 3 days. Hyphae were scraped off, and the edge of the fungal disc (with a diameter of 5 mm) was picked with a sterile toothpick and inoculated on PSA medium plate. Five replicates were conducted for each strain. After three days of growth in an incubator at 25 °C, the diameter of each colony was measured, and photographs were taken for documentation.

To observe the effect of *FgAsp* knockout on mycelial tips, a sterile coverslip was inserted at a 45° angle into 1/2 CM solid medium (Table S3). Mycelial plugs (with a diameter of 5 mm) of each strain were inoculated at the center of the plate. Five replicates were performed for each strain. After incubation at 25 °C, micrographs were obtained using fluorescence microscopy (Nikon Orthostatic Microimaging System NI-U) of hyphae present on approximately two-thirds of the coverslip.

Three mycelial plugs (with a diameter of 5 mm) of each strain were selected with a sterile toothpick, placed into 50 mL of carboxymethyl cellulose (CMC) liquid medium (Table S3), and incubated at 25 °C in a shaker (150 rpm) for 5 days. Then, 24 and 48 h media (containing sporulation structures) were taken, and the sporulation structures were observed under a microscope and photographed. After 5 days, the conidia was filtered with two layers of sterile paper and one layer of sterile filter cloth, and 10 μL of conidial suspension of each strain was aspirated onto a hemocytometer plate to calculate the rate of conidia production for each strain based on data from ten experiments using the five-point counting method (the conidia were counted in five areas on the hemocytometer (the area of the central grid and its four corners) using the 10× objective lens of the microscope). Conidia suspensions of the strains were subjected to a one-minute staining process with calcofluor white (CFW, Coolaber, BeiJing, 10 μg/mL) in the dark, after which the number of conidia exhibiting distinct septation patterns was counted. Each strain underwent a repetition of 100 conidia.

In order to observe the germination pattern of conidia, the conidia suspension was collected based on the sporulation rate statistics and transferred into 100 mL of yeast extract peptone dextrose (YEPD) liquid medium (Table S3). The suspension was cultivated at 25 °C with a rotation speed of 150 rpm for either 6 or 12 h. Observations were made using differential interference contrast microscopy.

### 2.6. Pathogenicity Tests

An appropriate quantity of sterilized wheat grains (Yangmai 158, 100 grains) was hydroponically cultured for 3 days. Meanwhile, conidia suspensions of PH-1, deletion, and complementation strains were collected and adjusted to 1–1.5 × 10^7^ conidia mL^−1^. The tip of the germ sheath was trimmed to a length of 1–2 mm, after which 5 μL of the conidia suspension was applied. This process was repeated for each strain with 15 replicated plants. Following 7 days of incubation at 25 °C and 90% humidity, the infection length was measured.

Conidia from different strains were collected similarly, with the concentration adjusted to 1.0–2.0 × 10^5^ conidia mL^−1^. A specific length of wheat leaf was cut and placed facing upwards on a PSA plate. Both ends of the leaf were sealed with PSA, and the middle section was gently scratched with the tip of a syringe needle. An aliquot of the conidia suspension (10 μL) was introduced to the scratched area. This process was repeated three times for each strain. After a five-day incubation period at 25 °C, the length of infection was measured.

For the inoculation of wheat spikes in the field, a conidia suspension with a concentration of 1.0 × 10^6^ conidia mL^−1^ was inoculated into the anthers of the central portion of the spike during the wheat flowering period. Subsequently, the spikes were sprayed with water and covered with plastic bags, which were removed after 48 h. After 14 days, disease incidence was recorded for all trials, and pictures were obtained. A total of 30 wheat spikes was inoculated for each strain, and the disease index for each strain was calculated according to standardized methods [31].

Three layers of sterile filter paper kept moist were placed in the petri dish, 5 mm of mycelium plug of each strain was placed on one end of four fresh corn whiskers, and the incidence was observed and recorded after 5 days of cultivation in an incubator at 25 °C. All experiments were repeated three times with three replicates at each time.

### 2.7. Sexual Reproduction Assays

After activation on PSA medium plates for 3 days, the mycelium was scraped off and the edge of the disk (5 mm in diameter) was inoculated onto carrot agar medium (Table S3) and incubated for 7 days at 25 °C in an incubator. After scraping off the aerial mycelium, it was incubated under a black light at 25 °C and observed daily. After the mycelium grew, 1% Tween 20 (Sinopharm, Shanghai, China) was used to fix the mycelium, and the incubation was continued for two weeks and recorded. Five replicates were performed for each strain. Then, the fungus cake was beaten with a 1 mL pipette tip, cut, placed vertically in disposable slides, and incubated at 25 °C for 24–48 h for observation of ascomycete spore eruption. Finally, the ascospore shells were scraped from the culture medium, placed on the slides and pressed, and the ascospore morphology observed under the microscope.

### 2.8. Determining DON Content and TRI Genes Expression Analysis

The concentration of DON was measured in cultures of the PH-1, Δ*FgAsp*, and CΔ*FgAsp* strains, which were cultured in liquid thioglycolate broth with indicator (TBI) medium (Table S3). The strains were grown in 20 mL of the medium at 28 °C in the dark with shaking at 120 rpm for 6 days. Supernatants from TBI cultures were performed using 8 mL of ethyl acetate. The extracts were then desiccated under a nitrogen stream, re-suspended in 1 mL of ethyl acetate, and analyzed using high-performance liquid chromatography coupled with tandem mass spectrometry (HPLC-MS/MS-6500, AB SCIEX, Framingham, MA, USA) to determine the DON content. The fungal cultures were obtained using the same method; RNA was extracted and reverse-transcribed, and the expression levels of TRI5, TRI6, TRI10, and TRI101 were determined [32].

After infection of the wheat spikes by *F. graminearum*, the amount of DON was determined. First, 5 grams of freeze-dried wheat powder was transferred to a 50 mL centrifuge tube, and 20 mL of an extraction solution (50% acetonitrile and 1% formic acid) in a 1:1 volume ratio was added. The mixture was vigorously vortexed for 5 min and then centrifuged at 3400× *g* rpm for 5 min. Then, 2 milliliters of the supernatant was taken and mixed with 0.2 g MgSO_4_, 0.1 g NaCl, 0.1 g Na_3_C_6_H_5_O_7_, and 0.1 g C18. The mixture was vortexed thoroughly for 1 min and then centrifuged at 3400 rpm for 5 min. The resulting supernatant was transferred to a 2 mL centrifuge tube, dried under nitrogen at room temperature, re-dissolved in 0.5 mL of mobile phase, and filtered through a 0.22 μm filter membrane, and the filtrate was used for chromatographic analysis in a 2 mL brown vial [33].

### 2.9. Sensitivity to External Stress and Transport of Different Sugars

The fungal strains were activated and placed on plates containing PSA alone or supplemented with 1.2 M NaCl, 1 M KCl, 0.2 M MgCl_2_, 1.2 M CaCl_2_, and 15 mM H_2_O_2_. Three replicate plates were performed for each strain. The plates were incubated at 25 °C in the dark, and the colony diameters were measured.

In MM media containing 3% sucrose, glucose, galactose, mannitol, or arabinose, blocks of PH-1, deletion mutants, and complementary mutants were placed on the media. Following a three-day incubation period at 25 °C, the colony diameters were measured.

## 3. Results

### 3.1. Bioinformatics Analysis

**Figure 2:**
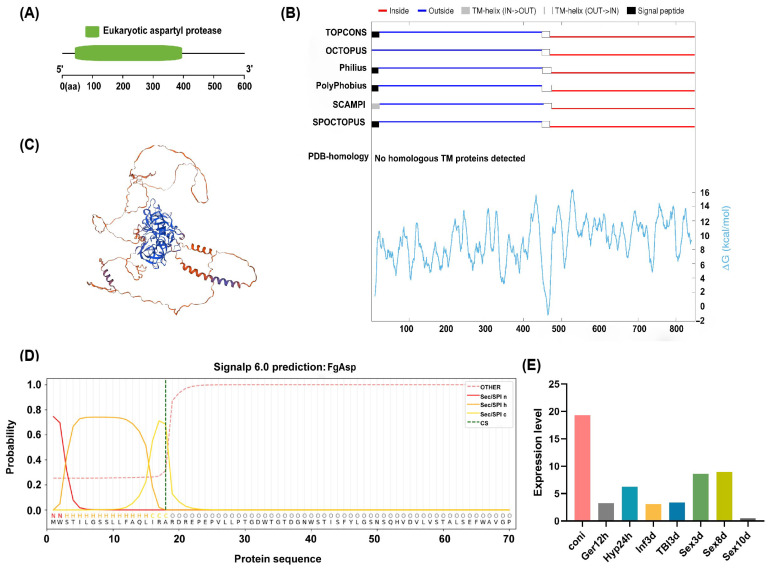
Description of *FgAsp*. (**A**) Conserved functional domain. (**B**) Identification of transmembrane domains. (**C**) Three-dimensional homology modeling. (**D**) Signal peptide prediction results. (**E**) Gene expression level of *FgAsp* in *F. graminearum*.

A conserved structural domain prediction of *FgAsp* was performed using the online tool InterPro, revealing that the gene contains a conserved structural domain named Eukaryotic aspartyl protease, located at amino acid positions 46 to 405 (Figure 2A). The online tool confirmed the presence of transmembrane domains in *FgAsp* (Figure 2B). Tertiary structure modeling indicated that *FgAsp* belongs to the membrane protein category (Figure 2C). Signal peptide prediction using SignalP-6.0 demonstrated that the *FgAsp* signal peptide has an 87.5% probability of being classified as SP (Sec/SPI). This analysis indicated a cleavage site between residues 22 and 23, confirming that the protein has a signal peptide (Figure 2D). The results suggest that the protein is a secreted protein containing aspartic protease.

Based on the gene expression data available at [27], the *FgAsp* gene was obtained from the *F. graminearum* genome database and drawn using Graphpad Prism 10.1.2to examine its expression patterns across various life stages of *F. graminearum* (Figure 2E). Expression analysis was conducted during the conidia stage (Coni), the mycelial stage, sexual development (3 and 8 days), and various infection stages on wheat. The results indicated that the *FgAsp* gene was expressed at all times, suggesting that the *FgAsp* gene plays a critical role in *F. graminearum*.

### 3.2. Deletion of the Gene FgAsp Is of Significant Importance for Mycelial Growth of F. graminearum

The DNA of the potential transformants obtained from the initial screening was used as a template for a crude PCR assay. Transformants were generated via protoplast transformation screening, with a transformation frequency of 14%. PCR electrophoresis results with four primers confirmed that 50% of the transformants were positive. Additionally, RT-PCR indicated that the Actin gene could be amplified in PH-1 and transformants strains, while the target gene was amplified only in the PH-1 strain, and it was not expressed in the knockout mutant Δ*FgAsp* (Figure S2B).

In the gene backup experiment, four complementary transformants were obtained, and PCR test results showed that all four had the correct size of the target gene band, indicating that the complementary strain CΔ*FgAsp* was successfully generated. One strain among the knockout and complementary transformants was randomly selected (Figure S2C).

**Figure 3:**
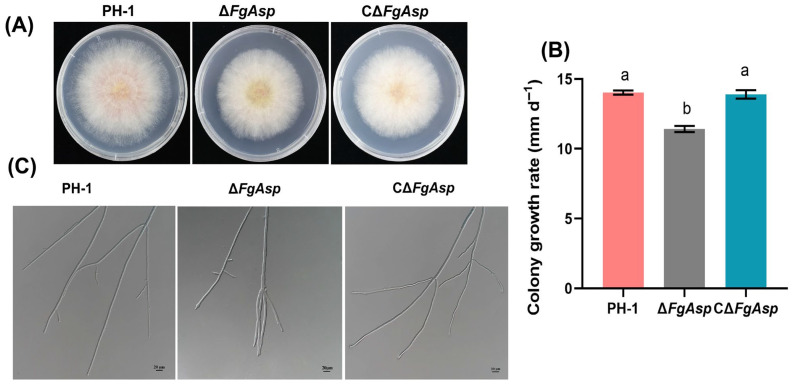
(**A**) Colony morphology of PH-1, Δ*FgAsp*, and CΔ*FgAsp*. (**B**) Growth rates of wild-type PH-1, Δ*FgAsp*, and CΔ*FgAsp* strains. (**C**) PH-1, Δ*FgAsp*, and CΔ*FgAsp* hyphal edge morphology. Scale bar = 20 μm. Means and standard errors were calculated using *t*-tests based on data from three independent biological replicates. Different letters indicate significant difference at the level of 0.05.

To investigate the effect of *FgAsp* on fungal growth, strains PH-1, Δ*FgAsp*, and CΔ*FgAsp* were inoculated on 20 mL of PSA medium. The growth rate of the CΔ*FgAsp* strain was statistically similar to that of PH-1, whereas Δ*FgAsp* exhibited a significantly reduced growth rate. Measurements of colony diameter and calculations showed that on PSA medium, the growth rate for PH-1 was 14.0 mm d^−1^, while that of Δ*FgAsp* was 11.4 mm d^−1^, indicating a 19% decrease in growth rate for Δ*FgAsp* compared with PH-1 (Figure 3A,B). Furthermore, microscopic observations of hyphal growth at the colony edges revealed abnormal tip branching of hyphae in Δ*FgAsp*, contrasting with the straight hyphae observed in PH-1 and CΔ*FgAsp* (Figure 3C).

### 3.3. FgAsp Gene Is Involved in the Production and Germination of Conidia

**Figure 4:**
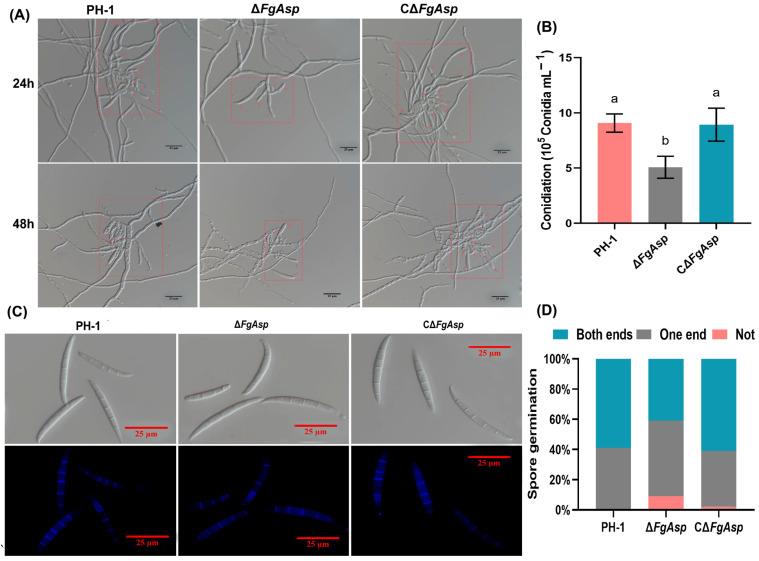
(**A**) Conidiophores of PH-1, Δ*FgAsp*, and CΔ*FgAsp*. The red arrows indicate the attached conidia on the conidial peduncle of each strain. Scale bar = 25 μm. (**B**) The sporogenesis rates of PH-1, Δ*FgAsp*, and CΔ*FgAsp*. Different lowercase letters a and b represent significant differences. (**C**) Statistics of the number of septa in conidia of PH-1, Δ*FgAsp*, and CΔ*FgAsp*. Scale bar = 25 μm. (**D**) Conidia germination statistics of PH-1, Δ*FgAsp*, and CΔ*FgAsp*.

The results of the study showed that the conidial peduncle produced by Δ*FgAsp* at 24 and 48 h was smaller in size compared with those of PH-1 and CΔ*FgAsp* (Figure 4A). After a five-day incubation period in CMC cultures, the production, number, and morphology of conidia were observed under the microscope. The Δ*FgAsp* strain produced 5.2 × 10^5^ conidia mL^−1^, reflecting a 42.1% decrease in yield compared with PH-1 (Figure 4B, Table S2). However, no significant differences were observed in the morphology of the conidia (Figure S3) or in the number of septa between Δ*FgAsp* conidia and PH-1 (Figure 4C). After 6 h of culturing in YEPD medium, the percentage of non-germinated Δ*FgAsp* conidia was 9%, with fewer conidia germinating at both ends compared with PH-1 and CΔ*FgAsp* (Figure 4D and Figure S4).

### 3.4. FgAsp Gene Is Crucial for Plant Infection

**Figure 5:**
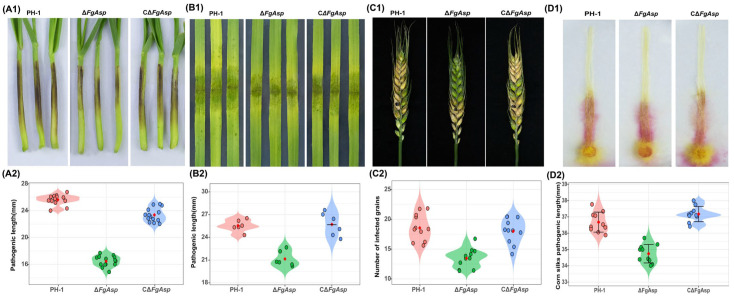
Pathogenicity and lesion length of PH-1, Δ*FgAsp*, and CΔ*FgAsp*: (**A1**,**A2**) Wheat coleoptiles, (**B1**,**B2**) wheat leaves, (**C1**,**C2**) wheat ears, (**D1**,**D2**) corn silks. The images above show the pathogenicity and lesion pictures, and the violin plot of lesion length is displayed below.

The pathogenicity of Δ*FgAsp* was evaluated in wheat coleoptiles, wheat leaves, field wheat spikes, and corn whiskers. Inoculation with the Δ*FgAsp* strain resulted in a reduction in pathogenicity of 9.2%, 4.4%, 5.1%, and 5.3%, respectively, compared with PH-1 (Figure 5A1–D2). Additionally, inoculation of conidia into wheat spikes during anthesis demonstrated that PH-1 and CΔ*FgAsp* caused typical disease symptoms, while Δ*FgAsp* exhibited reduced pathogenicity. Statistical analysis of the data, based on three independent biological replicates using the *t*-test method, showed that the disease indices for PH-1 and CΔ*FgAsp* were 4.1 and 4.2, respectively, 14 days after infestation of the wheat ears. In contrast, the average disease index for Δ*FgAsp* was significantly lower (2.6; Table S2). These findings suggest that the *FgAsp* gene influences the pathogenicity of *F. graminearum* by affecting mycelial growth and penetration ability, underscoring its crucial role in plant infection by the fungus.

### 3.5. Deletion of the FgAsp Gene Leads to Reduction in the Sexual Reproductive Ability

**Figure 6:**
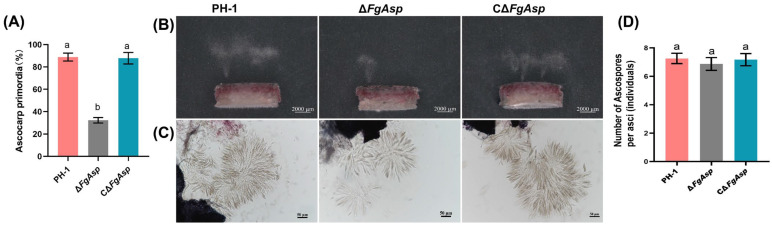
Sexual reproduction of PH-1, Δ*FgAsp*, and CΔ*FgAsp*: (**A**) Number of ascospores produced by sexual reproduction of PH-1, Δ*FgAsp*, and CΔ*FgAsp*. (**B**) Eruption of ascocarp primordia. Scale bar = 2000 μm. (**C**) Ascospores. Scale bar = 50 μm. (**D**) Number of ascospores per asci (individuals). Different lowercase letters a and b represent significant differences.

To investigate the impact of the *FgAsp* strain on sexual reproduction in *F. graminearum*, we examined its role in this process. The results demonstrated that after eight days of induction under black light on carrot agar, the Δ*FgAsp* strain produced a limited number of perithecium or ascocarp primordia, while the PH-1 strain generated a substantial quantity of mature black perithecium, some of which ejected yellowish perithecial beaks. Similar to PH-1, the CΔ*FgAsp* strain also produced a significant number of mature perithecium with yellowish perithecial beaks (Figure 6A and Figure S5A). Observation of the mycelial plugs from different strains under a dissecting microscope revealed that both the PH-1 and CΔ*FgAsp* strains exhibited widespread ejection, whereas the mutant lacking *FgAsp* showed only limited ejection (Figure 6B). After pressing the perithecium, the number of ascospores per ascum of Δ*FgAsp* was found to be non-significantly different compared with PH-1 and CΔ*FgAsp*. (Figure 6C,D and Figure S5B).

### 3.6. Production of DON Is Positively Regulated by FgAsp

**Figure 7:**
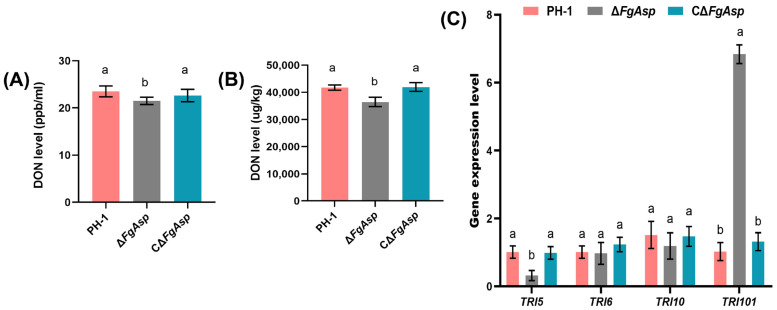
(**A**) DON toxin content in TBI medium of PH-1, Δ*FgAsp*, and CΔ*FgAsp*. (**B**) DON toxin content in wheat kernels of PH-1, Δ*FgAsp*, and CΔ*FgAsp*. (**C**) Expression levels of TRI gene clusters in PH-1, Δ*FgAsp*, and CΔ*FgAsp* after 6 days of TBI culture. Means and standard errors were calculated using *t*-tests based on data from three independent biological replicates. Different letters indicate significant difference at the level of 0.05.

To study the effect of *FgAsp* gene deletion on DON biosynthesis and production, DON content and gene expression level were assessed. Results showed that the DON content on Δ*FgAsp* TBI medium was lower than that of the PH-1 strain by 9.4% (Figure 7A), with a significant reduction of 12.5% in DON toxin content in wheat grains (Figure 7B). On 6-day-old TBI cultures, the expression levels of four TRI gene clusters indicated that the expression level of TRI5 in Δ*FgAsp* was significantly down-regulated, while the expression level of TRI101 was significantly up-regulated. These findings were consistent with the toxin detection results, suggesting that *FgAsp* plays a crucial role in the regulation of TRI gene clusters (Figure 7C).

### 3.7. FgAsp Positively Regulates Tolerance to Hydrogen Peroxide

In order to investigate whether the *FgAsp* gene plays a role in the ability of *F. graminearum* to regulate tolerance to exogenous stresses, the tolerance assays of each strain on PSA plates supplemented with salt ions were performed. Based on previous studies, 1.2 M NaCl, 1 M KCl, 0.2 M MgCl_2_, and 1.2 M CaCl_2_ were used.

**Figure 8:**
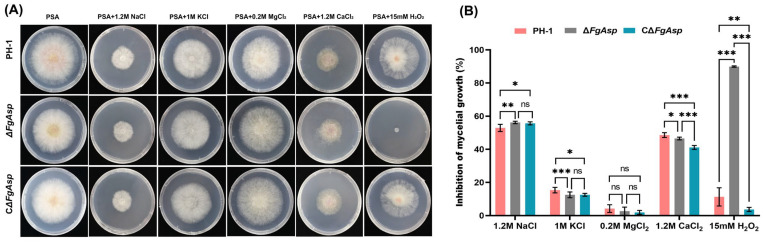
(**A**) Colony morphology of PH-1, Δ*FgAsp*, and CΔ*FgAsp* on PSA medium containing NaCl, KCl, MgCl_2_, CaCl_2_, and H_2_O_2_. (**B**) Stress growth inhibition rate analysis. Means and standard errors were calculated using *t*-tests based on data from three independent biological replicates. An asterisk (*) indicates a *p* value of less than 0.05, that is, the difference is significant at the 5% significance level. Two asterisks (**) indicate a *p* value of less than 0.01, that is, significant at the 1% significance level. Three asterisks (***) indicate a *p* value of less than 0.001, which is extremely significant at the 0.1% significance level. ns indicates no difference.

Compared with the wild-type strain PH-1, mycelial growth of the mutant strain did not show differences in the presence of MgCl_2_, but it showed slight differences in the presence of NaCl, KCl, and CaCl_2_. In the presence of NaCl, the wild-type strain PH-1 was 52.927 ± 0.987%, and the mutant mycelial growth inhibition was 56.208 ± 0.304%. In the presence of KCl, the wild-type strain PH-1 was 15.358 ± 0.688%, and the mutant mycelial growth inhibition was 12.518 ± 0.711%. In the presence of MgCl_2_, the wild-type strain PH-1 was 4.240 ± 0.956%, and the mutant mycelial growth inhibition was 2.688 ± 1.000%. In the presence of CaCl_2_, the wild-type strain PH-1 was 48.592 ± 0.591%, and the mutant mycelial growth inhibition was 46.550 ± 0.344%. These results imply that the *FgAsp* gene plays a role in the response of *F. graminearum* to sodium Na^+^, potassium K^+^, and calcium Ca^2+^ ion stress. Additionally, the strains’ sensitivities to oxidative H_2_O_2_ were tested. Interestingly, the mutant had nearly 100 percent inhibition of mycelial growth on hydrogen peroxide and was unable to grow aerial mycelium properly (Figure 8A,B). Overall, the results suggest that the *FgAsp* gene plays a key role in oxidative regulation, but it also may play a role in the osmotic regulation of *F. graminearum* (Figure 8A,B).

### 3.8. The FgAsp Gene Affects the Utilization of Arabinose

**Figure 9:**
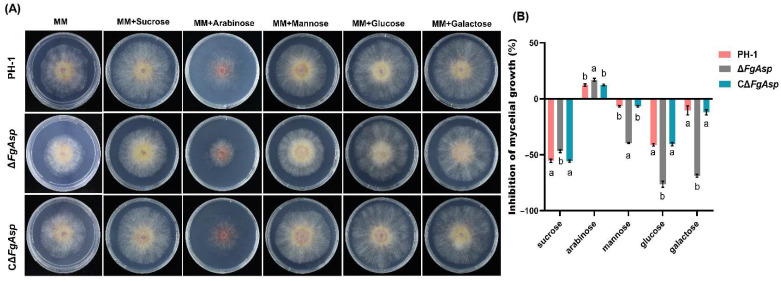
(**A**) Colony morphology of PH-1, Δ*FgAsp*, and CΔ*FgAsp* on PSA medium containing sucrose, arabinose, mannose, glucose, and galactose. (**B**) Analysis of different glycogen inhibition rates of wild-type PH-1, Δ*FgAsp*, and CΔ*FgAsp* strains. Data were tested by *t*-test, and error bars represent the standard deviation (SD). Different letters indicate a significant difference at the level of 0.05.

To investigate the impact of *FgAsp* on sugar utilization, the PH-1, Δ*FgAsp*, and CΔ*FgAsp* strains were cultured on MM media containing sucrose, arabinose, mannose, glucose, and galactose. The experiment demonstrated that after 3 days, the Δ*FgAsp* strain exhibited the most pronounced growth on MM media with arabinose; the diameter of the PH-1 strain was 54.5 mm, while the diameter of the Δ*FgAsp* strain was only 38.9 mm. At 5 days after inoculation, the colony diameter of Δ*FgAsp* on arabinose-containing MM media reached 55 mm, whereas it grew to 85 mm on media with the other sugars. It was determined that Δ*FgAsp* had the lowest utilization capacity for arabinose compared with PH-1 and CΔ*FgAsp* (Figure 9A,B).

## 4. Discussion

Aspartic proteases are a class of enzymes that catalyze the hydrolysis of proteins via aspartic acid residues. Aspartic acid is an important intermediate in the tricarboxylic acid cycle, participating in intracellular energy metabolism and ATP production; in the urea cycle, aspartic acid provides a nitrogen source for protein synthesis by participating in deamination reactions with amino acids. Secretory aspartic acid has been shown to play an important role in a number of biological processes, such as the growth and development of filamentous fungi and their virulence, and it is an indispensable class of protease for the basic life activities of fungal cells. It has proven effective in studies addressing drug resistance in *Candida albicans* infections [34]. In filamentous fungi, the secreted aspartic protease Ger1 has been shown to significantly affect spore germination in *Ustilago maydis* [35]. Similarly, *FolAsp* has been identified as a key factor contributing to the virulence of the pathogenic fungus *Fusarium spinosum*, the causal agent of tomato blight, while aspartic enzymes were found to be highly induced by the fungal cell wall in the secretory proteomic analysis of *Aspergillus hartshornii* [36]. Additionally, in *Aspergillus niger*, the deletion of four pepsin-like proteases led to an increase in the secretion level of heterologous laccase [37]. These studies suggest that secreted aspartic proteases play significant roles in the growth, development, and pathogenic processes of filamentous fungi. In the present study, the deletion of the secreted aspartic protease gene *FgAsp* resulted in defects in the asexual reproduction, sexual reproduction, and pathogenicity of *F. graminearum*. This finding aligns with previous important results regarding secreted aspartic proteases in pathogenic fungi and highlights potential targets for the development of fungicides to control wheat blast fungus. In the *C. albicans* SAP family (*SAP9* to *SAP1*), *SAP1*, *SAP2*, and *SAP3* are considered to be key genes for the complete pathogenicity process [38,39], and the *SAP8* and *SAP9* genes are associated with enhanced biofilm-forming capacity of *C. albicans*, which in turn affects virulence and pathogenicity process in *C. albicans* [16,40]. The MAP kinase or pathway cAMP positively regulates the expression of *SAP4*, *SAP5*, and *SAP6* genes associated with mycelial development by controlling two transcriptional activators, Cph1 and Efg1, while Efg1 also positively regulates *SAP1* and *SAP3* gene expression [41,42,43]. These findings suggest that there may be similarities in the mechanisms by which *FgAsp* affects plate colony morphology and complete virulence of *F. graminearum* on different carbon sources.

Regarding asexual reproduction, our results showed that the deletion of *FgAsp* caused abnormal mycelial tip branching in *F. graminearum* along with reduced mycelial edge growth on PSA, CM, and MM media. Backfilling with the *FgAsp* gene was able to restore the defects in the mutant to levels comparable to the wild type, leading us to conclude that secreted aspartic acid in *F. graminearum* may influence mycelial tip branching and, consequently, the rate of trophic mycelial growth. Additionally, we found that the strain exhibited a strong dependence on arabinose in various carbon source media. The deletion of *FgAsp* resulted in *F. graminearum* being unable to utilize arabinose as the sole carbon source for normal mycelial growth, while the *FgAsp* deletion mutant was less affected when glucose was used as the sole carbon source. Filamentous fungi typically preferentially select glucose and other easily utilized sugars, followed by carbon sources that may be detrimental to their growth and development [44]. Our results indicate that deletion of *FgAsp* amplifies the disadvantages of arabinose for *F. graminearum*. In terms of conidial development, we observed that the number of attached conidia on spore-producing structures formed within 24 and 48 h was lower in the mutant, which also exhibited a reduced germination rate compared with the wild-type and backfill strains during the same germination period. These findings suggest that *FgAsp* plays a role in the asexual reproduction of *F. graminearum*, a conclusion consistent with previous studies [36].

Ascospores formed during sexual reproduction play an important role in the infestation cycle of *F. graminearum* in the field. Huang et al. found that rice aspartic proteases are involved in the formation of male pollen in rice [45], and in *Cynara cardunculus*, aspartic proteases are involved in the entire reproductive development [46]. In our results, the deletion of *FgAsp* caused *F. graminearum* to be affected in its sexual reproduction, which was mainly manifested in the formation of fewer ascomycete shells and a reduced number of ascomycete spore eruptions in the *FgAsp* deletion mutant strains, but the number of ascomycete spores within a single ascomycete was not significantly different from that of the wild type. This suggests that aspartic protease is reported for the first time to be involved in sexual reproduction in *F. graminearum*.

Pathogenicity analysis is a crucial aspect of our study on phytopathogenic fungi. Our results demonstrated that deletion of *FgAsp* significantly reduced the virulence of *F. graminearum* on wheat germ sheaths, wheat leaves, wheat spikes, and corn silks. The DON toxin is a key virulence factor influencing the pathogenicity process of *F. graminearum*. We concluded that the mutants exhibited lower accumulated DON toxin content in both TBI-induced in vitro conditions and in field-infected wheat grains. To further elucidate the underlying mechanism, we found that key genes regulating DON synthesis, the TRI5, TRI6, and TRI10 gene clusters, were down-regulated, while the TRI101 gene in Δ*FgAsp*, which mitigates the autotoxicity of the DON toxin to the fungus, was up-regulated compared with PH-1 and CΔ*FgAsp* strains. These findings indicate that *FgAsp* positively regulates the synthesis and accumulation of the DON toxin and plays a significant role in the pathogenesis of *F. graminearum*.

In fungi, the upstream transcription factor that regulates the expression of secreted aspartic protease genes is known as PacC. This factor has been shown to be a determinant of virulence in both plant and animal pathogenic fungi while also playing important roles in meiosis, morphogenesis, and tolerance to NaCl-induced stress. Our results indicated that deletion of *FgAsp* rendered *F. graminearum* unable to respond effectively to salt ion-induced stress. To our surprise, deletion of *FgAsp* also resulted in increased sensitivity to hydrogen peroxide in *F. graminearum*, revealing a connection between secreted aspartic acid and oxidative stress.

In conclusion, in this study, we identified and elucidated that *FgAsp* is involved in *F. graminearum’s* mycelial growth, conidial development, sexual reproduction, pathogenicity, DON toxin metabolism, and stress tolerance.

## Data Availability

The original contributions presented in the study are included in the article/Supplementary Materials, further inquiries can be directed to the corresponding authors.

## Supplementary Materials

The following supporting information can be downloaded at: https://www.mdpi.com/article/10.3390/jof10120879/s1, Table S1. Primers used in this study; Table S2: Vegetative growth, conidiation, and virulence of *F. graminearum* strain; Table S3: The medium formula used in this study; Figure S1: Electrophoretic detection of the PCR-amplified fragment L1, L1, L2, H1, H2 and the fusion fragment LH1, HL2, M: DL2000 bp marker; Figure S2: PCR electrophoresis results of FgAsp knockout fragment and complementation fragments. (A) Lanes 1–4 are target gene, hygromycin, upstream homologous recombination, and downstream homologous recombination, respectively. (B) Lanes 5–8 are FgAsp gene knockout mutants as verified by RT-PCR. (C) Lanes 9–13 are PCR detection of Δ*FgAsp* gene complementation fragments, 15–18 are CΔ*FgAsp*, and 19 is PH-1. M: DL2000 bp marker; Figure S3: Conidia morphology of PH-1, Δ*FgAsp*, and CΔ*FgAsp*; Figure S4: Spore germination morphology of PH-1, Δ*FgAsp*, and CΔ*FgAsp*; Figure S5: (A) Ascocarp primordia. Scale bar = 500 μm. (B) Normal morphology of ascospores. Means and standard errors were calculated using *t*-tests based on data from three independent biological replicates. Different letters indicate significant difference at the level of 0.05.
