# Supplementary figures and images for: Functional Characterization of FgAsp, a Gene Coding an Aspartic Acid Protease in Fusarium graminearum
Source: J Fungi (Basel). 2024 Dec 17;10(12):879. doi: 10.3390/jof10120879 (PMC11679831; doi:10.3390/jof10120879)

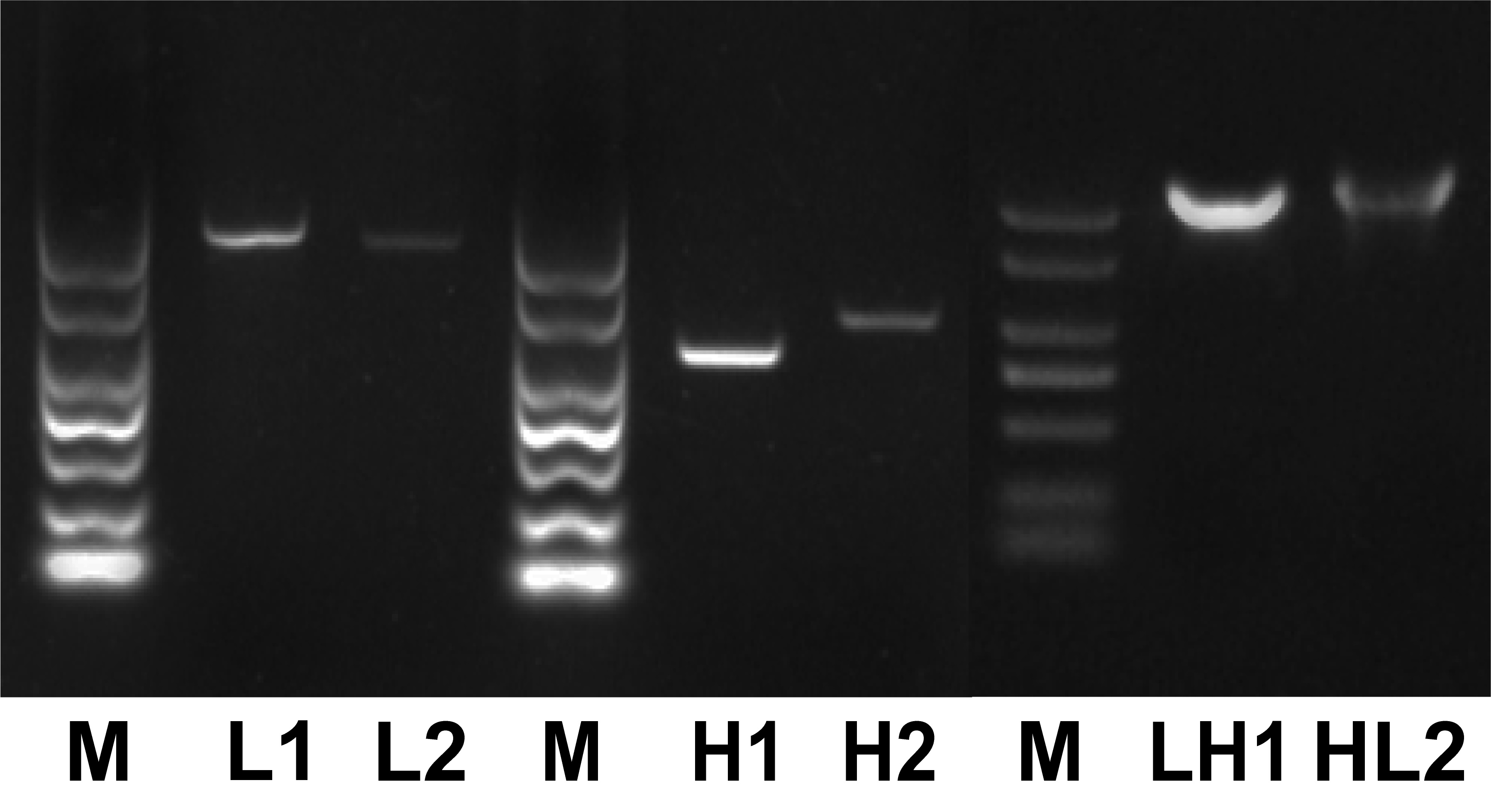

Supplement: Supplementary file 1 [file jof-10-00879-s001.zip › Figure S1.jpg]

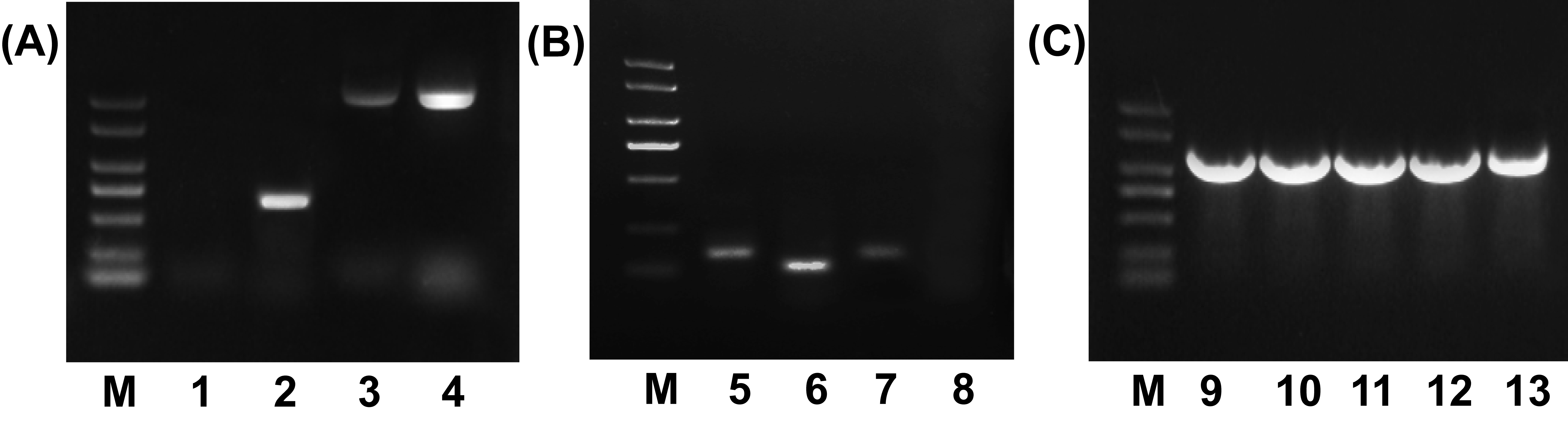

Supplement: Supplementary file 1 [file jof-10-00879-s001.zip › Figure S2.jpg]

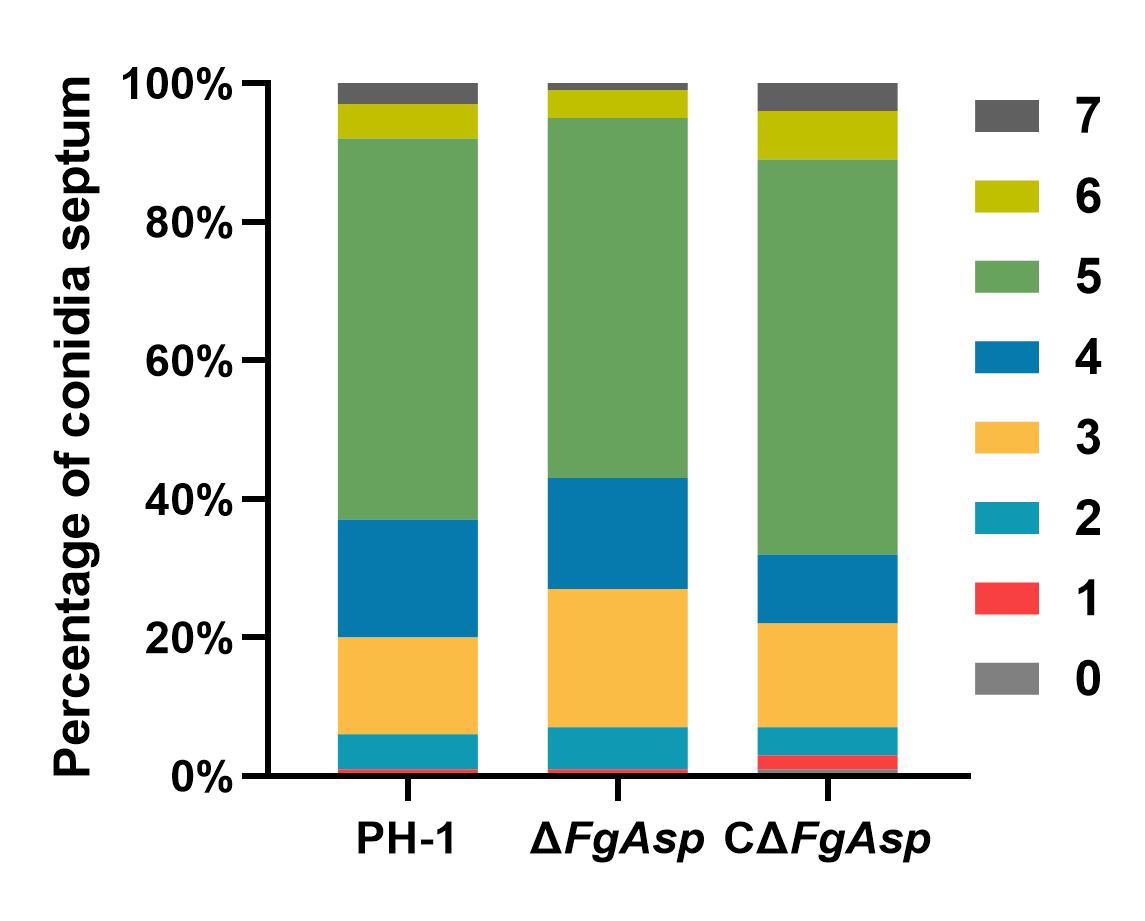

Supplement: Supplementary file 1 [file jof-10-00879-s001.zip › Figure S3.jpg]

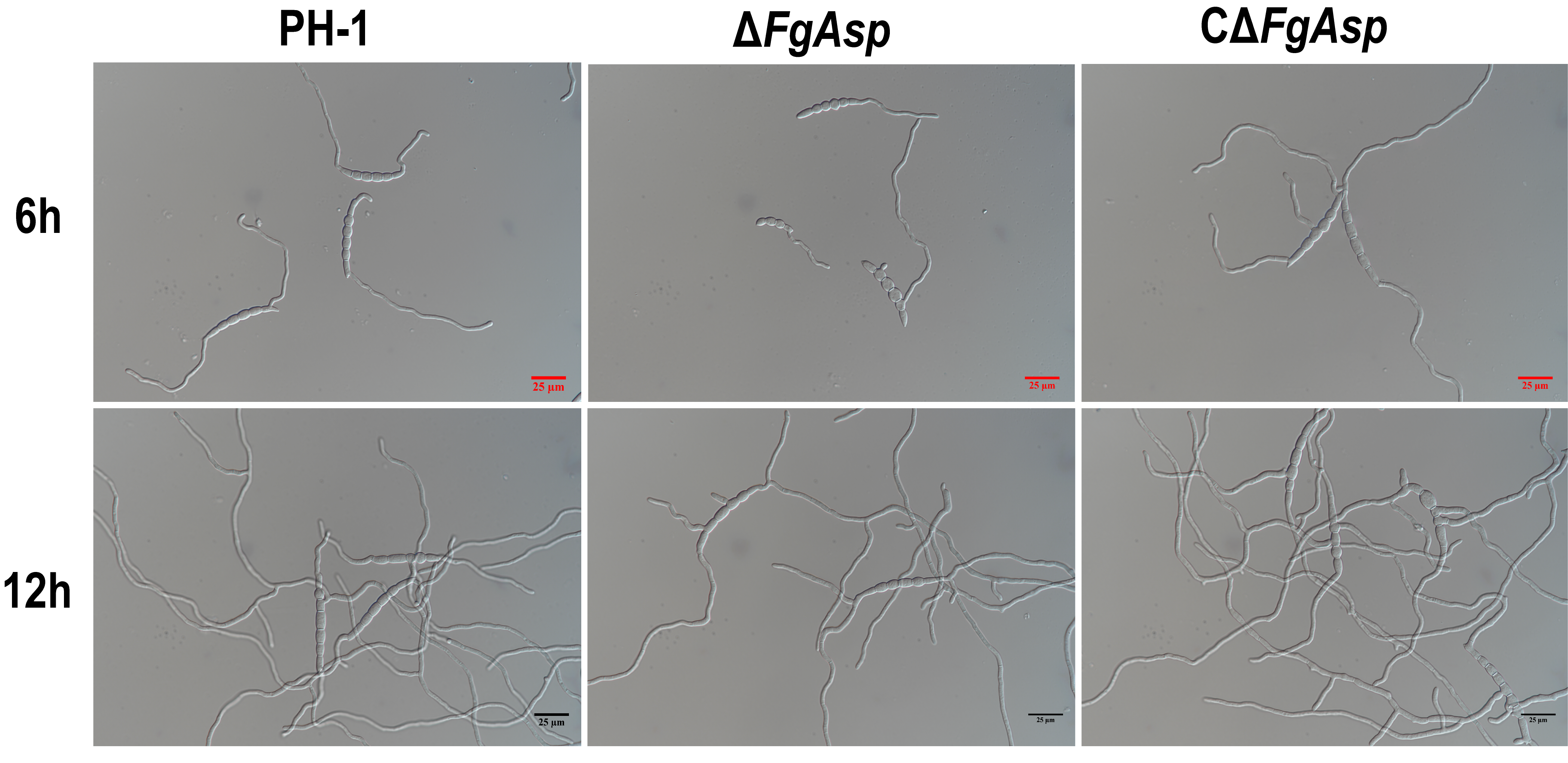

Supplement: Supplementary file 1 [file jof-10-00879-s001.zip › Figure S4.tif]

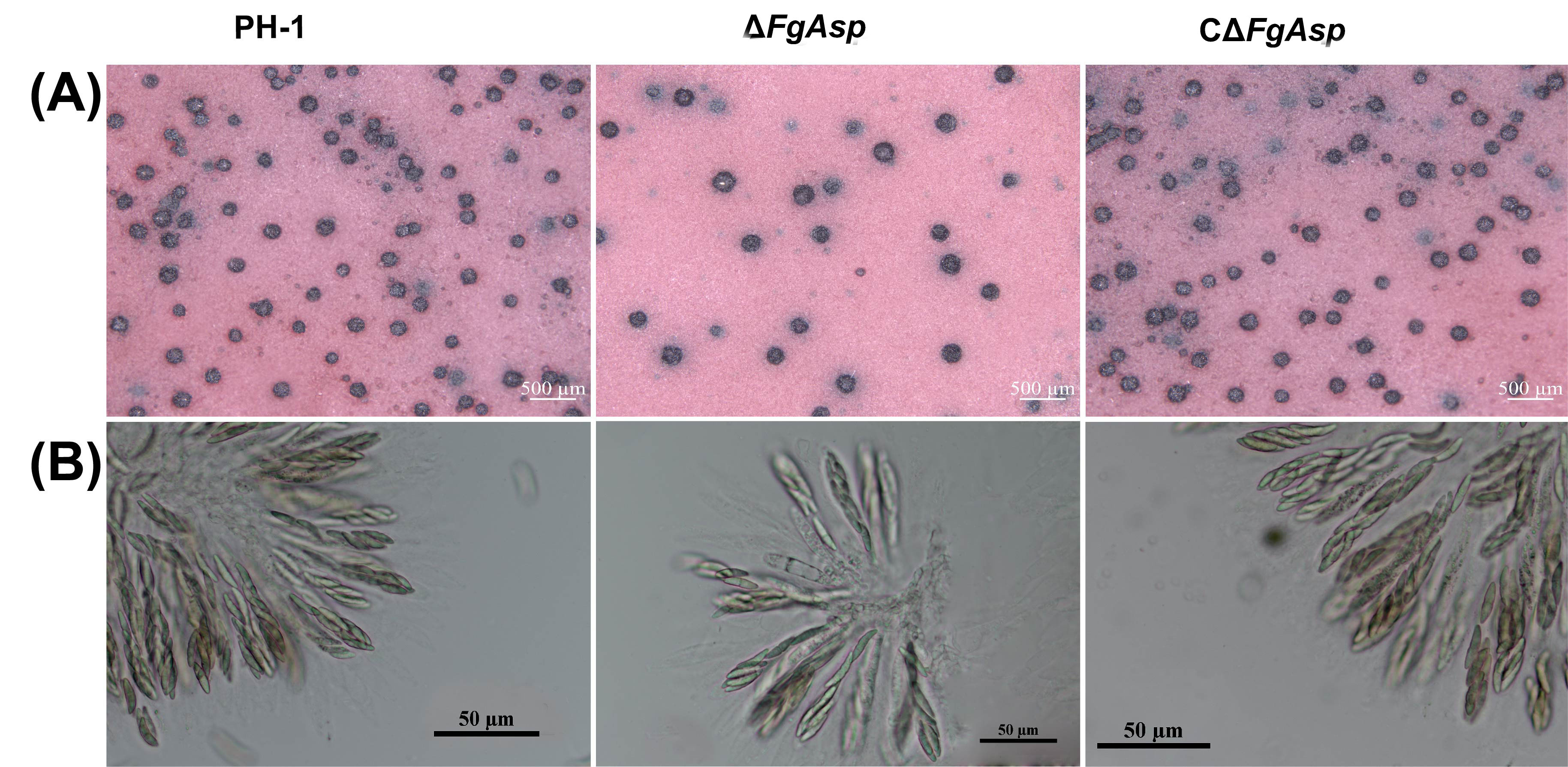

Supplement: Supplementary file 1 [file jof-10-00879-s001.zip › Figure S5.jpg]
